# Design and Methods for a Pilot Study of a Phone-Delivered, Mindfulness-Based Intervention in Patients with Implantable Cardioverter Defibrillators

**DOI:** 10.1155/2012/972106

**Published:** 2012-03-22

**Authors:** Elena Salmoirago-Blotcher, James Carmody, Gloria Yeh, Sybil Crawford, Lawrence Rosenthal, Ira Ockene

**Affiliations:** Division of Cardiovascular Medicine, University of Massachusetts Medical School, 55 Lake Avenue North, Room S3-855, Worcester, MA 01655, USA

## Abstract

*Background*. Meditation practices are associated with a reduction in adrenergic activity that may benefit patients with severe cardiac arrhythmias. This paper describes the design and methods of a pilot study testing the feasibility of a phone-delivered mindfulness-based intervention (MBI) for treatment of anxiety in patients with implantable cardioverter defibrillators (ICDs). *Design and Methods*. Consecutive, clinically stable outpatients (*n* = 52) will be screened for study eligibility within a month of an ICD-related procedure or ICD shock and will be randomly assigned to MBI or to usual care. MBI patients will receive eight weekly individual phone sessions based on two mindfulness practices (awareness of breath and body scan) plus home practice with a CD for 20 minutes daily. Patients assigned to usual care will be offered the standard care planned by the hospital. Assessments will occur at baseline and at the completion of the intervention (between 9 and 12 weeks after randomization). The primary study outcome is feasibility; secondary outcomes include anxiety, mindfulness, and number of administered shocks during the intervention period. *Conclusions*. If proven feasible and effective, phone-delivered mindfulness-based interventions could improve psychological distress in ICD outpatients with serious cardiovascular conditions.

## 1. Introduction

Sound evidence supports the value of meditation-based interventions in patients with cardiovascular disease. As far back as 1971, Wallace et al. [1] described the physiological characteristics of the “relaxation response” associated with meditative practices. These included a general decrease in sympathetic nervous system (SNS) activity, oxygen consumption, and blood lactate levels. Later studies comparing subjects practicing meditation with nonpractitioners found reduced endorgan sensitivity to catecholamines, possibly mediated by a lower percentage of functional lymphocyte beta-adrenergic receptors [2]. This overall reduction in the SNS activity may be beneficial in patients affected by cardiovascular disease as suggested by a number of studies of transcendental meditation [3–8]. Fewer studies, however, have explored the possible effect of mindfulness meditation interventions in this population [9–11].

The most widely available mindfulness training course is the mindfulness-based stress reduction (MBSR) program [12] created in the early eighties by Jon Kabat-Zinn. MBSR offers training in traditional mindfulness meditation adapted to a non-Buddhist, clinical context. While MBSR has shown positive results in a wide range of medical and psychological conditions, including anxiety and depression [13, 14] some features of the program, such as the need to attend eight weekly classes and the recommended duration of daily home mindfulness practice (usually 45 minutes), may constitute a barrier to participation for patients with severe chronic cardiovascular disease.

With this study, we sought to evaluate whether a new delivery method (phone delivery) would overcome some of the above-mentioned barriers to mindfulness training in patients with implantable cardioverter defibrillators (ICDs). This is a population with severe underlying cardiac conditions and considerable prevalence of psychological distress, with figures reaching up to 38% for clinically significant anxiety and depression [15, 16] for which mindfulness training may be beneficial. We describe here the design and methods of a pilot randomized clinical trial examining the feasibility of a mindfulness-based, phone-delivered intervention in a group of ICD patients. Secondary outcomes will include the preliminary estimation of efficacy of the intervention on anxiety and mindfulness scores.

## 2. Design and Methods

The “Coping with ICD study” (Clinicaltrials.gov NCT01035294) is a pilot randomized clinical trial designed to evaluate the feasibility of a phone-administered, mindfulness-based training program for the treatment of psychological distress in patients undergoing an ICD procedure, or reporting an ICD-related event (shocks).

### 2.1. Setting

The study will be conducted at the university campus of the UMass Memorial Medical Center (UMMMC), a tertiary care medical center located in Worcester, MA admitting more than 300 patients every year for ICD procedures.

### 2.2. Recruitment and Screening Procedures

Patients scheduled for an ICD-related procedure or who recently had an ICD-related event (shocks) will be screened for study eligibility within a month of the procedure/event. All potentially eligible patients will receive a letter inviting them to participate and asking them to call a dedicated phone number to communicate their possible interest. To ensure an unbiased presentation of the study, we developed a script of the first phone contact call. If the patient expresses interest, a screening visit will be scheduled.

Patients will be eligible if they meet the following criteria: age ≥21, ICD-related procedure or recent ICD shocks, ability to understand and speak English, and access to a telephone. Patients will be excluded from the study under the following conditions: inability/unwillingness to give informed consent, cognitive impairment, New York Heart Association (NYHA) functional class >III or Canadian Cardiovascular Society (CCS) angina class III or IV or otherwise clinically unstable, pending coronary bypass or heart transplantation, comorbid life threatening conditions, and ongoing severe depression or psychosis. The Blessed Orientation Memory and Concentration test [17] will be used to screen patients for cognitive impairment. Patients scoring ≥10 will be excluded from the study since mindfulness training requires a normal cognitive function and ability to focus the attention; cognitive impairment would limit the ability of the subjects to adequately participate in the intervention. Screening for ongoing depression and psychosis will be based on DSM criteria of major depressive disorder or psychosis as documented by the physician in the most recent medical evaluation. Once eligibility is confirmed, we will obtain informed consent in person. Since the study requires access to protected health information (PHI), a HIPAA authorization will be signed by each study subject in order to access his or her medical records. After baseline data collection is completed, participants will be randomly assigned to the intervention or to the control group.

### 2.3. Randomization

 The randomization sequence will be generated using STATA [18] “ralloc” command, which produces a sequence of group assignments randomly permuted in blocks of several sizes. Block sizes of 4 and 6 will be used in this study. A programmer will generate the random allocation sequence and upload the table containing the random sequence of group assignments to an Access database. Based on this table, the participant will be automatically assigned to a group by clicking the “Randomize” button. 

### 2.4. Follow-Up

 To maximize retention, patients in both study arms will receive a weekly phone call inquiring as to whether they had questions regarding their participation. When a participant misses an intervention session, he/she will be immediately contacted. After three missed contacts, nonresponders will be sent a letter encouraging them to discuss their status. Participants will not be expected to stop any of their usual support services, for example, professional counseling, support groups, or any antianxiety or antidepressant treatment.

The study protocol and the study materials were approved by the Committee for the Protection of Human Subjects at the University of Massachusetts Medical School (Docket H-13078).

### 2.5. Mindfulness-Based Intervention (MBI)

#### 2.5.1. Rationale

The study intervention will adapt elements of the MBSR [12] program (whose standard curriculum includes participation in eight weekly classes, lasting two and a half hours; an all-day retreat; practicing mindfulness and yoga exercises at home for 45 minutes/day) to the needs of patients with ICDs. Changes will include in person phone delivery versus in class delivery of the intervention; shortening of the duration of the training sessions and of the individual home practice; the exclusion of the all-day retreat and of yoga exercises.

Several conditions suggested the need for a modification of the standard MBSR program for ICD patients. (1) Driving is usually discouraged in ICD patients during the months immediately following ICD surgery [19] since up to 8% of patients experience a shock while driving [20]. This circumstance would limit attendance at regular MBSR classes. (2) Psychological distress is higher soon after ICD implantation [15], and it is important to start the intervention as close as possible to the ICD procedure to help alleviate symptoms when they are more intense. Since most MBSR programs are usually offered at discrete times, it may not be possible for many ICD patients to receive the intervention when it is most needed. (3) Physical activity may trigger arrhythmias and shocks [21], and it is often avoided by patients at this early stage for fear of ICD discharges. (4) The clinical condition of this population with severe underlying cardiovascular disease may limit their ability to attend classes and to practice mindfulness exercises for longer periods.

#### 2.5.2. Intervention Content

The conceptual background informing the intervention was guided by the “parsimonious” model recently proposed by Carmody [22]. Briefly, in this model mindfulness interventions are described as training in self-regulation of attention and recognition of the sensations, cognitions, and feelings that comprise daily experience. In the untrained individual, negative cycles of associated thoughts, sensations, and feelings are maintained by attention being absorbed in their content and/or meaning. For example, the presentation of a frightening thought generates an associational cycle in which the thought leads to unpleasant sensations of constriction. This negative cycle can begin with a thought, a feeling, or a sensation and is then self-maintained as long as the subject's attention remains engaged with the content of any of its components. In the process of mindfulness training, the subject learns to *notice* which component the attention is directed toward in any given moment and to *choose *to keep the attention where it is or to redirect it to another object, usually an arousal “neutral” object such as the sensations of breathing.

Consistently with our endeavor of adapting elements of the MBSR curriculum to meet the needs of ICD patients, the intervention's content will be simplified to include two basic components: (1) the body scan, a technique based on the cultivation of attention to bodily sensations and cognitions that would normally go unnoticed and (2) training in the awareness of the sensations of breathing. In addition, participants will be gradually taught to direct their attention to simple activities of daily life (such as eating and drinking), to sounds, visual objects, thoughts, and emotions and to recognize when their attention is no longer focused on that specific object of attention. At the final session, participants will practice “open awareness” in which they will be instructed to just notice which events (physical sensation, sound, visual object, and/or thought) their attention will be spontaneously drawn to from moment to moment. Patients will not receive additional materials usually provided to MBSR trainees in the form of poetry or other readings. In addition, lovingkindness (“metta” practice—a technique based on deliberately generating feelings of compassion, benevolence, and acceptance towards self and others) will not be a component of the study intervention. This technique was excluded because there is insufficient evidence for a benefit of such a practice on psychological well-being in patients with cardiovascular disease and because it would imply a different study hypothesis that deserves to be tested in a separate investigation.

#### 2.5.3. Intervention Format

 The study intervention will consist of eight phone-delivered, individual training sessions each lasting 30 minutes (Table 1). Twenty minutes will be spent on intervention delivery, and 10 minutes on questions, answers, and scheduling the next intervention. At the beginning of the study patients will receive an audio CD consisting of two different mindfulness practices, each lasting about 20 minutes, consistent with the techniques learned during each session with the instructor: track 1: sitting practice; track 2: body scan practice. After the delivery of the first intervention, participants will be encouraged to listen to the audio CD every day, at least once a day, and then throughout the study. The CD can be played using a regular CD player or a computer, and a portable CD player will be given to participants when needed.

**Figure 1:**
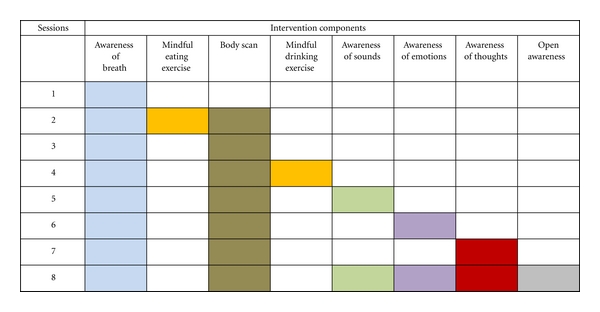
Overview of the study intervention component by session number. Each color indicates a different component of the intervention.

To ensure that the delivery of the intervention will be similar across instructors we developed a script of each session. Although instructors will not have to follow the script verbatim, they will be expected to follow the sequence indicated in the script.  Figure 1 shows the components of the intervention, each in a different color, and the session at which it was introduced. By looking at each row in this table it is possible to identify the content of each individual session.

### 2.6. Instructors

The instructors will be healthcare professionals and graduates of the Center for Mindfulness Professional Training Program with at least five-year experience in mindfulness training and a personal mindfulness practice. Prior to the study beginning, they will receive three hours of training, including a detailed review of the intervention script. We will hold bimonthly meetings to discuss any questions or difficulties that might be arising during the intervention sessions. Each patient will be trained by the same instructor throughout the intervention, and although not blinded to group assignment, instructors will be blinded to the study outcomes. At the end of each session, the instructors will complete an attendance form and a checklist in which duration and delivery of the intervention as specified in the intervention script as well as their perception of the patient's level of engagement during the session will be recorded. Patient's engagement will be evaluated immediately after each session and scored on a scale of 1 (completely unengaged) to 10 (extremely engaged). In order to monitor the provider's adherence to the protocol and the consistency of the delivery of the intervention across providers, each session will be digitally recorded by the instructor. Electronic copies of the attendance form, the checklist, and the MP3 file of the recorded session will be emailed weekly to the study manager.

### 2.7. Control Group: Usual Care (UC)

 Patients in the control group will receive the usual care provided by UMMMC to all ICD patients. Due to budgetary constraints, it was not possible to use an active control condition. To offset this limitation at least partially, patients in the usual care arm will receive a weekly phone call (duration: 5–10 minutes) that, although not designed to offer a specific intervention, will be aimed at addressing patients' possible concerns regarding their health or the ICD. If such concerns presented, the patient will be advised to contact his/her physician or nurse at the electrophysiology clinic. This phone call will also help to equalize the amount of study contact between study arms.

### 2.8. Study Assessments

Data collection will be performed at the baseline interview immediately after consent is provided, and nine weeks after enrollment once the intervention is completed.

#### 2.8.1. Primary Outcome: Feasibility

Feasibility metrics include eligibility and recruitment rates, retention rates, intervention adherence rates, treatment fidelity, and patient's experience with the intervention. Recruitment metrics include number of screened and eligible patients, number of eligible patients who refused to participate, and reasons for refusal. Retention measures will be the number of subjects who dropped out or were lost to followup and reason(s) for dropping out. Adherence metrics include number of sessions attended and total time spent in mindfulness practice in hours over the intervention period. In addition, the time spent engaging in each separate technique will be collected. Mindfulness practice will be self-reported by means of a daily diary that patients will receive at the consenting visit, and will be instructed to mail them back using prestamped envelopes. A similar diary was successfully used in a study [23] of the effect of mindfulness training on hot flashes in menopausal women. Treatment fidelity (developed following Treatment Fidelity Workgroup guidelines) [24] will be both self-reported by the instructors (by means of a checklist to be completed at the end of each session) and objectively evaluated by reviewing a random sample (10%) of all recorded sessions, and will be defined as the average of the ratio between the number of objectives achieved versus the number of objectives planned for each session, multiplied by 100, calculated from the checklist form.

#### 2.8.2. Secondary Outcomes

**Mindfulness**

Baseline and postintervention mindfulness scores will be measured using the Five Facets of Mindfulness (FFM) questionnaire [25], an instrument derived from a factor analysis of questionnaires measuring mindfulness in daily life. It consists of 39 items, exploring different aspects of mindfulness: observing, describing, acting with awareness, nonjudging of inner experience, and nonreactivity to inner experience. Each item is rated on a Likert scale ranging from 1 (never or very rarely true) to 5 (very often or always true). The FFM has shown good internal consistency [25].

**Anxiety**

Anxiety will be measured using the Hospital Anxiety and Depression Scale, [26] a 14-item self-administered questionnaire with two subscales measuring anxiety and depression, with higher scores indicating greater psychological morbidity. A cutoff point of 8 is usually recommended to screen patients for clinically significant depression and anxiety [26–28]. A correlation between 0.6 and 0.8 has been reported between the HADS and other commonly used questionnaires for anxiety and depression such as the Beck Depression Inventory and the State-Trait Anxiety Inventory [27], and its validity has been confirmed both in hospital settings and primary care medical practice [29]. Furthermore, the HADS offers the advantage of focusing on cognitive symptoms of anxiety instead of physical symptoms; this is particularly useful in cardiac patients where symptoms of the underlying cardiac disease may be similar or identical to those resulting from somatic manifestations of anxiety.

**Number of Shocks**

The number of delivered shocks (if any) will be abstracted from the electronic version of the follow-up visit 9 weeks after enrollment. While patients can receive care in other centers during the follow-up period, ICD-specific follow-up visits are mostly performed at UMMMC, thus limiting the chance of missing important information about ICD functioning and arrhythmic events.

**Covariates**

Information will be collected on demographics (age, gender, ethnicity, education, marital status, and financial status), type of ICD, indication for and time from the ICD procedure or shocks, prior history of anxiety and depression, ejection fraction and cardiac functional status, hospital readmissions during the study period, ongoing medications as well as other relevant data such as physical activity, use of other complementary medicine treatments, and life events (i.e., death or illness of spouse or relative) during the study period.

### 2.9. Data Sources

 Demographic data, physical activity, use of other complementary/alternative therapies, anxiety, and mindfulness scores will be obtained from self-administered standardized questionnaires. Medical history, including past history of anxiety or depression, prescription of psychotropic medications and antiarrhythmic drugs, indication for ICD implantation, functional class, and number of shocks/arrhythmic episodes and hospital readmissions will be abstracted directly from the medical record. Study questionnaires will be administered by in-person interview at baseline and by phone interview at week 9. Questionnaires delivery via phone interview (following intervention) was preferred to mailing of questionnaires because response rates tend to be higher using the telephone as compared with mailed surveys [30]. Furthermore, the study population will likely include older individuals who may have difficulties reading the surveys. There is evidence that for some people, particularly those of low literacy and education, telephone interview is less intimidating.

### 2.10. Data Management

Daily management of study activities will be facilitated by the use of Microsoft Access 2007 tracking system software. Scores from study questionnaires will be immediately calculated, copied into abstraction forms together with other relevant study variables, and then entered into STATA software [18]. The study database will be kept on a server at the University of Massachusetts Medical School, with multiple levels of password protection ensuring data security. All statistical analyses will be performed using STATA version 10 statistical software [18].

### 2.11. Statistical Analysis

Descriptive statistics will be used to describe retention and adherence indices; a graphical examination of the distribution of the continuous variables will be used to assess the need for transformation and to show patterns (e.g., whether the amount of self-reported daily mindfulness practice shows preferential “patterns” of practice). Correlations between duration of individual mindfulness practice and baseline characteristics such as age, gender, education, and severity of cardiac illness will be evaluated using Spearman's correlation. We will assess the preliminary estimates of effect sizes of the MBI intervention on pre-/postintervention differences in mindfulness and anxiety scores using multivariate linear regression models (shown here for FFM scores): *y* (pre-/postintervention difference in FFM scores = *β*_0_ + *β*_1_ TX (0 control, 1 treatment) + *β*_2_ age + *β*_3_ gender (0,1) + *β*_4_ baseline FFM *⋯* + *ε*.

### 2.12. Sample Size

Our planned sample size (*n* = 52, 26 for each arm) has been calculated (Table 2) using hypothetical estimates of effect size on anxiety (secondary outcome), based on data from two studies using HADS anxiety scores as the outcome variable in similar patients [31, 32]. Based on our previous experience with mindfulness-based interventions in menopausal women and bone-marrow transplant candidates, a potential loss of 20% of patients may be expected during the study, and consequently we will need to enroll 52 patients in order to leave 42 for the final analysis.

## 3. Discussion

The purpose of this study is to evaluate whether a mindfulness-based behavioral intervention delivered over the phone and adapted to the needs of ICD patients would be feasible and acceptable to these individuals. Since the proposed intervention will involve several changes from the traditional MBSR program, the question arises of whether such changes are legitimate. Since its inception in the early eighties the MBSR program has been modified several times to meet the needs of hospitalized patients [33] or to minimize the time commitment required [34]. Carmody et al. [35] found no association between the number of class hours employed in published trials of MBSR and the effect sizes for measures of psychological distress. Furthermore, different psychological interventions, including cognitive behavioral therapy, [36–45] have been successfully implemented over the phone. Given this, it seems reasonable to hypothesize that mindfulness training could be delivered over the phone, and, in fact, phone delivery may have an important impact on retention: the attrition rate reported in a meta-analysis of studies evaluating the effect of telephone-administered psychotherapy on symptoms of depression was only 7.6%.

This study presents some limitations, which for the most part reflect its pilot nature and budgetary constraints. First, we did not have the financial and personnel resources to plan for the recruitment from additional clinical centers to achieve an ethnically diverse population. For similar reasons we will not be able to have an active control comparison group. Under ideal conditions, a three-arm randomization to a nurse education intervention, a mindfulness intervention, and usual care would probably be the optimal design. A usual care comparison group does not control for the possible effect deriving from the interaction with the instructor, independently of the intervention administered. Second, study assessments will occur only before and after the intervention. Further data collection points (i.e., at six months and one year) would provide useful information about a possible long-term effect of the intervention on anxiety and possibly on the number of shocks. However, this pilot study is a very preliminary exploration of the possible effects of a mindfulness-based intervention on anxiety in cardiac patients and specifically, in ICD-implanted patients. It seems that evidence of a short-term effect is warranted before the analysis can be carried on further. Third, study participants will not be blinded. This is a common limitation of behavioral interventions; however, assessors will be blinded to patients' treatment allocation status and the instructors will be blinded to study outcomes. Finally, the individual mindfulness practice will be self-reported. With adequate funding, it would be possible to develop techniques to track the amount of time that each participant listens to the study CD, such as the device used by Bauer-Wu et al. [33] or by posting each recorded session on a dedicated intervention website and then tracking the time that each participant is logged onto the website.

In conclusion, this project has potentially great significance considering the prevalence of anxiety in this population (up to 40%) [15] and the increased number of candidates for ICD implantation due to a broadening of ICD indications to include primary prevention of sudden death [46]. To date, there have been no published studies of mindfulness-based interventions in ICD patients. A mindfulness-based intervention, adapted to this group of patients, is relatively inexpensive and, once learned, could be easily self-administered by the patient. If proven feasible and effective, it could positively impact the psychological well-being and the quality of life of these patients.

## Figures and Tables

**Table 1:** Characteristics of the study intervention.

Component	Duration	Objectives/content	Strategies/materials
Screening visit	10 minutes	(i) Patient receives general instructions about the intervention by the study manager (ii) Patient receives study CD player if needed, intervention CD, and mindfulness diary	Study CD player Intervention CD Mindfulness diary

Phone sessions (8)	30 minutes	(i) Instructor checks on patient ability to practice specific mindfulness technique(s) taught during previous session(s) (ii) Instructor inquires about symptoms/side effects (iii) Instructor guides patient in mindfulness exercise and receives feedback from patient (iv) Instructor and patient develop goals for next session (v) Instructor encourages participant to practice mindfulness technique with help of study CD (specifies track, 20′every day) (vi) Instructor arranges next phone session (vii) Instructor completes intervention checklist (viii) Instructor reports problems to study manager	Intervention checklist Digital recorder for session recording by the instructor

**Table 2:** Sample size calculations^§^.

Measure	Instrument	Definition	Hypothesized mean (SD) control group	Hypothesized mean (SD) intervention group	Sample size (total)
Anxiety	HADS	Mean differences between baseline and postintervention	3.0 (2.2)	5.1 (2.6)	42

^§^Null hypothesis Ho = mean difference in pre- /post-HADS score in intervention group = mean difference in pre- /post-HADS score in control group; *α* (two-tailed) = 0.05 and 1− *β* = 0.8.
